# Identifying fibroblast growth factor receptor genetic alterations using RNA‐based assays in patients with metastatic or locally advanced, surgically unresectable, urothelial carcinoma who may benefit from erdafitinib treatment

**DOI:** 10.1002/cjp2.163

**Published:** 2020-04-18

**Authors:** Songbai Wang, Mike Burgess, Christopher Major, Alistair English, Maranna Sweeney, Arndt Hartmann

**Affiliations:** ^1^ Janssen Research & Development, LLC, Oncology Diagnostics Raritan NJ USA; ^2^ QIAGEN Manchester Ltd., Product Development Manchester UK; ^3^ University of Erlangen, General Pathology and Pathological Anatomy Erlangen Germany

**Keywords:** companion diagnostic, erdafitinib, fibroblast growth factor receptor (FGFR), urothelial carcinoma

## Abstract

Erdafitinib, a pan‐fibroblast growth factor receptor (FGFR) inhibitor received accelerated approval from the US Food and Drug Administration (FDA) for locally advanced or metastatic urothelial carcinoma (mUC) in adult patients with specific *FGFR3/2* genetic alterations who progressed during or after ≥1 line of prior platinum‐containing chemotherapy (PCC), including within 12 months of neoadjuvant or adjuvant PCC. Concordance between the clinical trial assay (CTA) used in a phase 2 study and QIAGEN's *therascree*n® FGFR kit (a two‐step, multiplex, real‐time, RT‐PCR assay), the FDA‐approved companion diagnostic (CDx) with erdafitinib, was evaluated in this bridging study. Study samples included 100 CTA‐confirmed FGFR‐positive samples from 100 erdafitinib‐treated mUC patients, plus 200 CTA‐confirmed FGFR‐negative samples from the phase 2 study. The primary objective was met if the lower bound of 95% CI of objective response rate (ORR) in CDx‐confirmed patients with FGFR alterations was >25%. Demographics were similar between the bridging study and CTA‐screened patients. In total, 292 of 300 samples (97.3%) with valid CDx results showed high analytical concordance versus CTA (percent agreement [95% CI]: positive percent agreement, 87.2 [79.0; 92.5]; negative percent agreement, 97.0 [93.5; 98.6]; overall percent agreement, 93.8 [90.5; 96.1]). Investigator‐assessed ORR in the 81 CDx‐identified, erdafitinib‐treated patients who tested positive for both assays was 45.7% (95% CI: 35.3%; 56.5%) versus 40.4% (95% CI: 30.7%; 50.1%) for CTA and met the criteria for primary objective. High ORR and clinical concordance to CTA suggest that QIAGEN's CDx can reliably select mUC patients who would potentially benefit from erdafitinib treatment.

## Introduction

Treatment of metastatic urothelial carcinoma (mUC) in patients who are cisplatin‐ineligible or cisplatin‐refractory represents a significant unmet medical need. Gene expression and sequencing studies have improved our understanding of molecular subtypes of UC and revealed signature genomic alterations that are potentially targetable, paving the way for personalized medicine 1. Genetic alterations in fibroblast growth factor receptor (FGFR) and the related signaling axis are associated with increased cell proliferation and migration, angiogenesis and anti‐apoptotic mechanisms and occur in approximately 15% of mUC with a preponderance in upper UC 2, 3, 4. The luminal I molecular subtype of UC, in particular, is characterized by *FGFR3* mutations, *FGFR3* fusion and an upregulation of FGFR mRNA and protein expression 5, 6.

Erdafitinib, a potent pan‐FGFR (FGFR1–4) tyrosine kinase inhibitor, has shown antitumor activity in several cancer cell lines that was associated with inhibition of downstream FGFR signaling 7. In a phase 2 study (BLC2001; NCT02365597), erdafitinib demonstrated a clinically meaningful objective response rate (ORR) and an acceptable safety profile, emerging as a first‐in‐class treatment for patients with surgically unresectable or mUC harboring *FGFR* mutations/fusions 8. Based on these results, erdafitinib was granted accelerated US Food and Drug Administration (FDA) approval for locally advanced or mUC in adult patients with susceptible *FGFR3/2* genetic alterations, whose disease progressed during or following ≥1 line of platinum‐containing chemotherapy (PCC), including within 12 months of neoadjuvant or adjuvant PCC 9. Concurrently, the FDA also approved the *therascreen®* FGFR RGQ (Rotor‐Gene Q MDx instrument) Reverse transcription (RT)‐polymerase chain reaction (PCR) kit, developed by QIAGEN Manchester Ltd., for use as a companion diagnostic (CDx) with erdafitinib to detect *FGFR* genetic alterations and guide patient selection for the approved therapeutic indication 10.

CDxs are critical for the implementation of personalized medicine and enable appropriate use of the paired therapy by accurately identifying patients who would most likely benefit from the targeted treatment 11, 12. The FGFR inhibitor Clinical Trial Assay (CTA), a RT‐PCR assay developed by Janssen, USA, and performed by Almac Diagnostics (Craigavon, UK) was used to determine the *FGFR* alteration status in patients enrolled for the erdafitinib phase 2 study. Given the differences in assay design and instrument platform between the CTA and QIAGEN's CDx assay, a bridging study was designed to compare the clinical performance of the CDx versus the CTA, establish agreement between two assays and evaluate clinical efficacy outcome of erdafitinib in patients with locally advanced or mUC identified with *FGFR* alteration using the CDx.

## Materials and methods

The *therascreen®* FGFR kit (CDx) developed by QIAGEN is a qualitative *in vitro* diagnostic test for the detection of four‐point mutations (R248C, S249C, G370C, and Y373C) and five fusions (TACC3_V1, TACC3_V3, BAIAP2L1, CASP7, and BICC1) in the *FGFR2/3* genes. It is a two‐step, multiplex, real‐time RT‐PCR test designed to detect *FGFR* alterations in RNA derived from formalin fixed paraffin embedded (FFPE) UC tissue samples archived during the BLC2001 study 8.

### Study design and population

Details of study design and eligibility criteria of the BLC2001 study have been reported previously 8. The primary objective of this study was to evaluate the ORR (i.e., complete response [CR] + partial response [PR]) of the selected dose regimen of erdafitinib in patients with histologically confirmed mUC or locally advanced, surgically unresectable UC 8. Eligible patients were either chemo‐refractory (failed ≥1 prior chemotherapy) or chemotherapy‐naïve, and ineligible for cisplatin‐based therapy. Patients were also required to have select *FGFR3* mutations or *FGFR2/3* fusions based on evaluation of appropriate tumor tissue samples. Molecular screening was performed centrally at Almac Diagnostics using the CTA, a two‐step RNA‐based PCR assay designed to detect *FGFR* alterations in FFPE tissue. All FFPE samples were banked by Almac during the entirety of the BLC2001 study 8.

**Figure 1:**
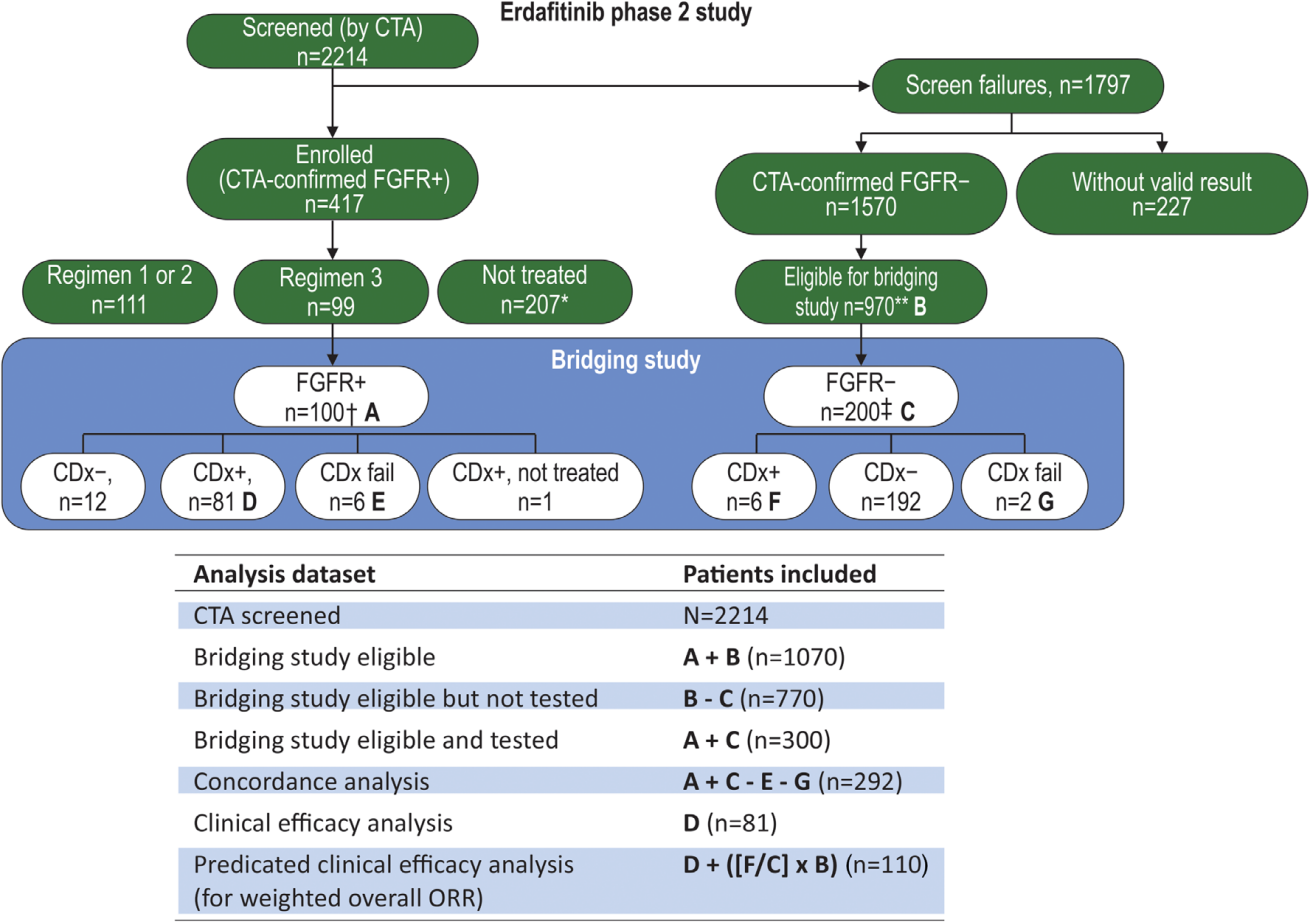
Study design and sample selection. *Includes 1 untreated patient in regimen 3. **n = 600 samples were not eligible for the bridging study (reasons: received before November 28, 2015, no consent for bridging testing, insufficient samples or passing sample store limit). ^†^Includes 1 FGFR+ patient who was not treated but was eligible for the bridging study. ^‡^320 patients were randomly selected and then 120 patients were removed due to a change in the selecting protocol. CDx, companion diagnostic assay; CTA, clinical trial assay; FGFR, fibroblast growth factor receptor; ORR, objective response rate.

Archived, residual FFPE UC tissue samples from a subset of enrolled patients (regimen 3: erdafitinib 8 mg once daily, pharmacodynamically guided up‐titration to 9 mg once daily) and CTA‐confirmed *FGFR*‐negative (CTA−) patients screened during the BLC2001 study were used for this bridging study (Figure 1). Eligible samples were evaluated retrospectively using the investigational CDx FGFR kit and the results were compared to the CTA results. All tests were performed at Almac Diagnostics.

The relevant independent ethics committee or institutional review board approved the study protocol, including the use of archived tumor tissue. Informed consent forms were collected at the Drug Study Investigator sites and were not provided to the device investigator but were available for verification if requested by study monitors.

### The CDx: testing procedure

The FGFR kit comprised the RNeasy DSP FFPE kit, the QIAGEN RGQ and the Rotor‐Gene Assay Manager (RGAM) software package for automatic analysis and reporting of the results. The detection was based on selective amplification of nine *FGFR2/3* alterations in RNA extracted from FFPE UC specimens using the RGQ system.

#### RNA extraction and sample handling

RNA extraction was performed using the RNeasy DSP FFPE kit on 4 to 5 μm thick FFPE sections with a tumor area between 100 and 500 mm^2^. Macrodissection was performed if necessary to attain a minimum of 80% tumor area. All steps outlined in the RNeasy DSP FFPE kit protocol were performed at room temperature (15 to 25 °C). Once extracted the RNA samples could either be tested immediately with the RT‐PCR kit or stored at −90 to −65 °C and a maximum of five freeze–thaw cycles were permissible.

For the bridging study, two independent RNA extractions were performed for each patient sample. If sufficient tissue was not available, residual CTA‐derived RNA was used. After extraction, the two RNA samples from the same patient were pooled and the RNA quantified by spectrophotometry using the absorbance at 260 nm. Each pooled RNA sample was divided into two equal aliquots (A1 and A2). The first aliquot (A1) was tested using the FGFR kit at Almac Diagnostics. The second aliquot (A2) was stored at −100 to −65 °C and shipped to Horizon Discovery (Cambridge, UK) for use in the accuracy validation study. Samples with RNA quantity below minimum requirements (18 ng/μL) were not used in the accuracy study and were stored at Almac Diagnostics.

#### Reverse transcription

The FGFR RGQ RT‐PCR kit provides high cDNA yields for sensitive detection of all target transcripts. It uses an RNA template and a blend of primers complementary to 3′ and 5′ ends of the RNA to produce the cDNA. The reverse transcriptase enzyme synthesizes the first strand of cDNA, which is used as input into the PCR. The process of RT was performed by incubating the normalized RNA (250 ng) sample (A1) with a master mix containing the primer mix and reverse transcriptase enzyme provided with the FGFR kit. The resulting cDNA sample was then subjected to real‐time PCR using the four real‐time PCR multiplex reaction mixes.

#### Real‐time PCR on the Rotor‐Gene Q MDx (US) instrument

The FGFR RGQ RT‐PCR kit contained four ready‐to‐use PCR mixes that include HotStarTaq DNA Polymerase and PCR buffer. A specific set of oligonucleotide primers within each of four reaction mix reagents was used to amplify target *FGFR* mutations and fusions. For each of the two mutation mixes, a blocking oligonucleotide specific for the wild‐type region of each target was included to increase amplification specificity. Independent mutation‐specific TaqMan oligonucleotide probes detected the specific *FGFR* targets. The RGQ and associated software were used to control the real‐time PCR and to determine the *FGFR* alteration status of the clinical samples. The RGAM software provided general functionality including PCR run setup and thermal cycling control, as well as management of data, results, assay profiles, and system configuration.

#### Analysis

Based on predetermined analytical cycle threshold value (*C*_T_ value: the PCR cycle at which the fluorescence from a particular reaction crosses a threshold value), the RGAM software qualitatively determined the *FGFR* alteration status of the samples and reported which samples were positive for the indicated *FGFR* alteration. The run controls (positive and negative template controls) were assessed to ensure that *C*_T_ values were within the internal control acceptance criteria. Samples were classified as target positive if the *C*_T_ was less than or equal to the cut‐off for the target assay.

### Bridging study assessments

Representativeness analysis was performed to determine if the study sample selected for the bridging study was representative of the CDx intended use. Demographics and tumor characteristics for the patients tested in the bridging study were compared with the rest of patients who were eligible but not tested in the bridging study.

Assessment of analytical concordance between the CDx and CTA was based on estimation of positive percent agreement (PPA), negative percent agreement (NPA), and overall percent agreement (OPA). PPA was calculated as the proportion of CDx‐confirmed *FGFR*‐positive (CDx+) samples given that the samples were CTA‐confirmed *FGFR*‐positive (CTA+); NPA was calculated as the proportion of CDx‐confirmed *FGFR*‐negative (CDx−) samples given that the samples were CTA−; and OPA was calculated as the proportion of agreement between CDx and CTA among all samples tested. In addition, to determine the overall accuracy of the CDx, discordant and concordant samples were tested and compared with a validated reference standard, droplet digital PCR (ddPCR). An orthogonal method was developed based on ddPCR technology. The ddPCR FGFR system constituted a two‐step nucleic acid RT and multiplex ddPCR test on the QX200TM Droplet Digital™ PCR System. The assay was developed and validated by Horizon Discoveries for the specific detection of the nine *FGFR* targets of interest. A contrived model of *in vitro* transcription RNA for each target spiked into a wild‐type universal RNA background was initially used to develop and optimize the assay. As described previously, the second aliquot (A2) was tested at Horizon Diagnostics using the RT‐ddPCR assay.

The primary clinical efficacy objective was to estimate the ORR (defined as proportion of patients with CR or PR in all treated patients) of regimen 3 in patients who were CDx+. The secondary objective was to estimate the ORR in a subgroup of erdafitinib‐treated chemotherapy‐relapsed/refractory patients who were CDx+.

### Statistical analysis

Demographics and baseline characteristics were summarized descriptively. For representativeness analysis, the *P* values were calculated based on two group *t*‐test for continuous measures and chi‐square test for category data. In the analysis of concordance, the PPA, NPA, and OPA, along with the two‐sided 95% confidence interval (CI), were calculated using the CTA as the reference method. For the purpose of power analysis in evaluation of concordance, 90% PPA and 95% NPA were expected. The accuracy study acceptance criteria were met if the lower limit of the two‐sided exact 95% CI for PPA for overall mutation status was ≥85% and the lower limit of the two‐sided exact 95% CI for the NPA for overall mutation status was ≥90%.

Percentage ORR was summarized along with 95% CI. The primary objective was met if the lower bound of the 95% CI for the observed ORR in CDx+ patients was >25%. The overall ORR in all patients was calculated by taking the weighted average of ORR for positive discordant (CDx+/CTA−) and positive concordant (CDx+/CTA+) patients; however, since CTA− patients were not included in the BLC2001 study, a range of hypothetical ORR values (i.e., 0%, 25%, 50%, 75%, and 100% of observed ORR in positive concordant patients) were used. Bootstrapping was performed to calculate 95% CI of the weighted ORR.

## Results

### Study sample selection

In total, 300 CTA‐screened samples comprising 100 CTA+ samples (from regimen 3, *n* = 99 erdafitinib‐treated) and 200 CTA− samples from the BLC2001 study were eligible for analysis in the bridging study (Figure 1). Of these 300 samples, 292 (97.3%) yielded a valid result, 3 (1.0%) had insufficient FFPE and CTA‐extracted RNA for testing and 5 (1.7%) samples were invalid as they did not meet PCR testing quality control criteria.

### Demographics and baseline characteristics

Demographics between the bridging study patients and CTA‐screened patients were similar (Table 1). The mean (SD) age of the bridging study patients was 66.9 (9.74) years with a preponderance of men (74.7%). The majority of samples were collected from the primary tumor origin (85%) with 73.8% viable cells and 69.5% tumor area. A comparison of the demographic profiles did not show any significant (*p* < 0.05) difference between patients tested and not tested in the bridging study, except for ethnicity and percent viable cells.

**Table 1:** Demographics and baseline characteristics

Characteristic	CTA screened, *n* = 2214	Patients tested, *n* = 300	Patients not tested, *n* = 770	*P* value*
Age, years, mean (SD)	66.6 (9.87)	66.9 (9.74)	66.6 (10.04)	0.66
Sex, *n* (%)				0.82
Male	1687 (76.2)	224 (74.7)	580 (75.3)	
Female	527 (23.8)	76 (25.3)	190 (24.7)	
Race, *n* (%)				0.11
White	1454 (65.7)	204 (68.0)	545 (70.8)	
Black	23 (1.0)	6 (2.0)	4 (0.5)	
Asian	312 (14.1)	28 (9.3)	79 (10.3)	
Other	425 (19.2)	62 (20.7)	142 (18.4)	
Ethnicity, *n* (%)				0.02
Hispanic/Latino	35 (1.6)	10 (3.3)	7 (0.9)	
Not Hispanic/Latino	1707 (77.1)	226 (75.3)	596 (77.5)	
Unknown/not reported	471 (21.3)	64 (21.3)	166 (21.6)	
Region, *n* (%)				0.16
North America	350 (15.8)	53 (17.7)	101 (13.1)	
Asia	346 (15.6)	34 (11.3)	95 (12.3)	
Europe	1518 (68.6)	213 (71.0)	574 (74.5)	
Tumor area, %, mean (SD)	61.9 (30.37)	69.5 (24.30)	69.4 (23.47)	0.95
Viable cells, %, mean (SD)	64.9 (25.18)	73.8 (20.58)	69.7 (22.98)	0.01
Site of tumor collected, *n* (%)				0.07
Primary	1871 (84.5)	255 (85.0)	691 (89.7)	
Metastatic	341 (15.4)	45 (15.0)	78 (10.1)	
Unknown	2 (0.1)	0	1 (0.1)	

* *P* value (tested versus not tested) calculated based on two‐group *t*‐test for continuous measures and chi‐square test for category data. CTA, Clinical Trial Assay; SD, standard deviation.

### Concordance analysis between CTA and CDx

Estimation of percent agreement (95% CI) between CDx and CTA, with CTA as the reference method, demonstrated good analytical concordance for the 292 samples with valid CDx results (PPA, 87.2 [79.0; 92.5]; NPA, 97.0 [93.5; 98.6]; OPA, 93.8 [90.5; 96.1]) (Table 2).

**Table 2:** Concordance analysis for CDx and CTA (reference) *FGFR* gene mutation screening methods

	CTA (reference)		
CDx	FGFR+	FGFR−	Total
FGFR+, *n*	82	6	88
FGFR−, *n*	12	192	204
Total, *n*	94	198	292
Percent agreement, % (95% CI)			
PPA	87.2 (79.0; 92.5)		
NPA	97.0 (93.5; 98.6)		
OPA	93.8 (90.5; 96.1)		

CDx, companion diagnostic assay; CTA, clinical trial assay; FGFR, fibroblast growth factor receptor; NPA, negative percent agreement; OPA, overall percent agreement; PPA, positive percent agreement.

### Accuracy validation

A total of 306 valid results were generated using the ddPCR *FGFR* assay: 104 *FGFR*‐positive samples, 202 *FGFR*‐negative samples. A high level of concordance was observed between the CDx and reference ddPCR *FGFR* assay (percent agreement [95% CI]: PPA, 99.04 [94.76; 99.98]; NPA, 97.52 [94.32; 99.19]; OPA, 98.04 [95.78; 99.28]) that met the criteria for the accuracy study (Table 3).

**Table 3:** Accuracy study: ddPCR results

		ddPCR		
		Negative	Positive	Total, *n* (%)
CDx	Negative	197	1	198 (64.71)
	Positive	5	103	108 (35.29)
	Total, *n* (%)	202 (66.01)	104 (33.99)	306 (100.00)

Measure of agreement	Frequencies	Percent agreement	95% CI
OPA	300/306	98.04	95.78; 99.28
PPA	103/104	99.04	94.76; 99.98
NPA	197/202	97.52	94.32; 99.19

CDx, companion diagnostic assay; ddPCR, droplet digital polymerase chain reaction; NPA, negative percent agreement; OPA, overall percent agreement; PPA, positive percent agreement.

### Clinical efficacy analysis

Investigator‐assessed ORR in the 99 erdafitinib‐treated patients who were CTA+ in the regimen 3 of the BLC2001 study was 40.4%. Overall, 81 of these 99 patients also tested positive on the CDx assay. The investigator‐assessed ORR (95% CI) was 45.7% (35.3%; 56.5%), meeting the criterion for primary objective (Table 4). The ORRs by *FGFR* alterations ranged from 0.0% for *FGFR3‐BAIAP2L1* fusion (*n* = 1) to 63.6% for the *FGFR3‐Y373C* mutation (*n* = 11).

**Table 4:** Investigator‐assessed ORR in erdafitinib‐treated patients who were FGFR+ by both CDx and CTA assays

	All treated patients		
	FGFR+ patients, *n*	Patients with response, *n*	ORR, % (95% CI)
Overall	81	37	45.7 (35.3; 56.5)
Point mutations	68	34	50.0 (38.4; 61.6)
FGFR3‐R248C	13	7	53.8 (29.1; 76.8)
FGFR3‐S249C	42	19	45.2 (31.2; 60.1)
FGFR3‐G370C	3	1	33.3 (6.1; 79.2)
FGFR3‐Y373C	11	7	63.6 (35.4; 84.8)
Fusions	18	6	33.3 (16.3; 56.3)
FGFR2‐BICC1	0	0	—
FGFR2‐CASP7	0	0	—
FGFR3‐BAIAP2L1	1	0	0 (0; 79.3)
FGFR3‐TACC3_V1	14	5	35.7 (16.3; 61.2)
FGFR3‐TACC3_V3	5	1	20.0 (3.6; 62.4)

CDx, companion diagnostic assay; CTA, clinical trial assay; FGFR, fibroblast growth factor receptor; ORR, objective response rate.

The results for weighted overall ORR further supported the efficacy concordance between CDx and CTA. Even for a hypothetical ORR in the positive discordant patients at 25% of the observed ORR, the weighted overall ORR was 41.7% (95% CI: 31.5%; 52.2%) and met the primary efficacy objective (Table 5).

**Table 5:** Weighted ORR in CDx+ patients

	Hypothetical ORR values in patients with CDx+/CTA, %	Weighted ORR, % (95% CI)
100% × observed ORR in CDx+	45.7	45.7 (34.8; 56.5)
75% × observed ORR in CDx+	34.3	44.4 (33.7; 54.9)
50% × observed ORR in CDx+	22.8	43.0 (32.6; 53.5)
20% × observed ORR in CDx+	11.4	41.7 (31.5; 52.2)
0% × observed ORR in CDx+	0.0	40.4 (30.5; 50.8)

CDx, companion diagnostic assay; CTA, clinical trial assay; ORR, objective response rate.

In total, 87 of the 99 treated patients in the BLC2001 study were chemo relapsed/refractory and the investigator assessed ORR in these CTA‐enrolled patients was 40.2% (95% CI: 29.9%; 50.5%). Sixty‐nine of these patients tested CDx+ in the bridging analysis and 32 of 69 had an investigator assessed ORR (46.4% [95% CI: 35.1%; 58.0%]).

## Discussion

The *therascreen®* FGFR RGQ RT‐PCR kit developed by QIAGEN could reliably identify patients with *FGFR3* point mutations and *FGFR2/3* fusions who are eligible for erdafitinib treatment. Overall, the bridging study population was demographically similar to the CTA‐screened BLC2001 study population 8. Significant differences in ethnicity and viable cells could be attributed to the large sample sizes for these analyses and were not regarded as clinically meaningful. The high PPA, NPA, and OPA between the CDx assay and the CTA are in line with the expected level of performance and demonstrate a good level of analytical concordance between the two assays. In addition to the measurement of agreement, the accuracy of the CDx RT‐PCR assay was demonstrated against the sensitive ddPCR test that further validates the robust clinical performance of the FGFR kit. High ORR was observed with erdafitinib treatment in patients with *FGFR* gene events identified by the CDx and the primary objective of >25% ORR was met, consistent with the BLC2001 study. This conclusion is further supported by imputation analysis which demonstrated that, when all patients with CDx+/CTA− were assumed to be nonresponsive to erdafitinib, there was no negative impact to the weighted ORR.

FGFR is an attractive target for several solid malignancies including UC, the sixth most prevalent type of cancer in the United States 13. The intratumor heterogeneity of the *FGFR3* alterations in UC determines sensitivity to specific targeted chemotherapy and therefore it is important to employ accurate identification tests to detect these driver mutations and to maximize potential benefits of FGFR‐targeted therapies 1, 2. CDx tools are therefore an indispensable component of personalized medicine and should be designed and validated in close collaboration during the clinical development of the paired therapy 11. Diagnostic errors due to inaccurate molecular tests have shown serious consequences such as patient misclassification and incorrect therapy resulting in negative treatment outcomes and undermining the benefits of personalized molecular therapies 14.

Overall, the performance measures validated in this bridging study should be interpreted in the context of the study population and design.

After receiving breakthrough therapy designation in March 2018, erdafitinib was granted accelerated approval by the US FDA in April 2019 10. The expedited development of erdafitinib has been complemented with the development of a robust CDx, the QIAGEN FGFR kit, which is also the first FDA‐approved CDx assay to detect actionable *FGFR* alterations in patients with locally advanced or mUC. Several FGFR inhibitors such as FGF401 (FGFR4 inhibitor), AZD4547, and BGJ398 (both FGFR1–3 inhibitors) have been investigated in phase 1 and 2 studies 15. Different methods including fluorescence *in situ* hybridization (FISH), immunohistochemistry, quantitative real‐time PCR, and next‐generation sequencing (NGS) have been used in these studies for the detection of *FGFR* mutations for screening and selection of patients 16, 17, 18. In a phase 2 study of dovitinib (a nonspecific FGFR inhibitor), *FGFR3* mutations were assessed using a custom‐designed SNaPshot assay (ThermoFisher Scientific, Waltham, MA, USA) for all common mutations in *FGFR3* coding exons including exons 7, 10, and 15. The assay was performed on representative transurethral resection of bladder tumor specimens 19. In a phase 1 study of combination treatment with everolimus and pazopanib in genomically selected patients, deep‐targeted NGS was used to screen exonic DNA of 400 known cancer genes, including *FGFR* alterations 20. Although there are no standard recommendations on assays to detect *FGFR* mutations, with more specific FGFR inhibitors such as erdafitinib coming into clinical practice, targeted techniques and multiplex testing such as NGS would increase the likelihood of identifying actionable *FGFR* mutations. However, concordance of NGS with PCR‐based methods must be established before using NGS in routine practice. Thus, the availability of a validated CDx for the approved therapeutic indication could potentially increase access to the test and assist clinicians to efficiently select suitable patients who would benefit the most from erdafitinib therapy. In conclusion, the findings of high analytical concordance, accuracy of diagnosis and consistent detection of clinical response benefit to erdafitinib in the intended patient population from the BLC2001 study supports the clinical performance of QIAGEN's *therascreen®* FGFR RGQ RT‐PCR kit as a CDx for erdafitinib.

## Author contributions statement

SW, MB, CM, AE, MS, and AH were involved in the study design, data collection, analysis, and interpretation. SW and AE were the project statisticians and oversaw data analysis and interpretation. All authors met the ICMJE criteria and those who fulfilled the criteria are listed as authors. All authors had access to the study data, provided direction and formal review of the manuscript, and made the final decision about where to publish these data. All authors contributed toward drafting and revising the paper and agreed to be accountable for all aspects of the work.
